# TAFRO Syndrome

**DOI:** 10.4274/balkanmedj.galenos.2020.2020.3.30

**Published:** 2020-08-11

**Authors:** Hirohisa Fujikawa, Makoto Araki

**Affiliations:** 1 Department of Medical Education Studies, International Research Center for Medical Education, Graduate School of Medicine, The University of Tokyo, Tokyo, Japan; 2 Department of Internal Medicine, Suwa Central Hospital, Nagano, Japan

A 54-year-old male patient presented with a 3-day history of fever. Physical examination revealed bilateral edema of lower extremities. Laboratory investigations showed a hemoglobin level of 136 (normal 135-176) g/L, platelet count of 62x10^9^/L (normal 131-362x10^9^/L), lactate dehydrogenase level of 136 (normal 130-240) U/L, alkaline phosphatase level of 428 (normal 100-350) U/L, creatinine level of 0.67 (normal 0.6-1.0) mg/dL, and C-reactive protein level of 22.23 (normal 0-0.30) mg/dL. Serum immunoglobulins were within normal ranges, and monoclonal protein was not detected in serum and urine. Autoimmune workup was negative, and infectious workup was negative, including human herpesvirus-8 (HHV-8) and human immunodeficiency virus. The level of vascular endothelial growth factor (VEGF) was elevated (175, normal <38.3 pg/mL). Computed tomography demonstrated mild splenomegaly (Figure 1A). Fluorine-18 (18F)-fluorodeoxyglucose positron emission tomography-computed tomography revealed pleural effusion and systemic mild lymphadenopathy with increased 18F-fluorodeoxyglucose uptake (Figure 1B). Three weeks after admission, fever lasted. Blood tests showed anemia (hemoglobin level of 97 g/L), deteriorated thrombocytopenia (platelet count of 28x10^9^/L), and acute kidney injury (creatinine level of 1.57 mg/dL).

A biopsy of the right axillary lymph node revealed atrophic germinal centers with enlarged endothelial cell nuclei, expanded interfollicular zone, endothelial venule proliferation, and a relatively small mature plasma cell number (Figure 1C). Bone marrow biopsy demonstrated hypercellular marrow, megakaryocyte hyperplasia, and mild reticulin fibrosis (Figure 1D). We diagnosed thrombocytopenia, anasarca, fever, reticulin myelofibrosis, and organomegaly (TAFRO) syndrome. The patient was treated with prednisolone (60 mg/day), which was gradually tapered and discontinued after approximately 3 years of treatment. The patient has no recurrence. Written informed consent was obtained from the patient.

The TAFRO syndrome is a newly recognized disease concept (1) and is considered to be an uncommon subtype of idiopathic multicentric Castleman disease (iMCD), which is negative for both polyneuropathy, organomegaly, endocrinopathy, monoclonal gammopathy, and skin changes syndrome and HHV-8 (2). The pathogenesis of TAFRO syndrome has yet to be fully understood, but it is assumed to be a cytokine storm including VEGF and interleukin-6 (3). TAFRO syndrome develops acutely or subacutely and is frequently life threatening, whereas non-TAFRO iMCD usually progresses chronically (2).

No optimal treatment has been established, but corticosteroids are the most commonly used first-line therapy. Other choices are immunosuppressants, immunomodulators, and cytotoxic chemotherapy. Because late relapses are not infrequent, long-term follow-up is warranted for patients with TAFRO syndrome.

## Figures and Tables

**Figure 1:**
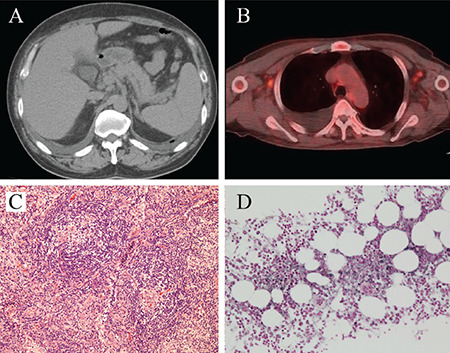
**A-D.** (A) Computed tomography showing a slightly enlarged spleen. (B) Fluorine-18 (18F)-fluorodeoxyglucose positron emission tomography-computed tomography showing pleural fluid and small lymphadenopathy with augmented 18F-fluorodeoxyglucose uptake. (C) Biopsy of the right axillary lymph node showing atrophic germinal centers with enlarged endothelial cell nuclei, expanded interfollicular zone, dense endothelial venule proliferation, and a few mature plasma cells (hematoxylin and eosin stain, original magnification x10). (D) Bone marrow biopsy showing hypercellular marrow and megakaryocytic hyperplasia along with reticulin fibrosis (silver stain, original magnification x20).
